# Two-dimensional nanovermiculite and polycaprolactone electrospun fibers composite scaffolds promoting diabetic wound healing

**DOI:** 10.1186/s12951-022-01556-w

**Published:** 2022-07-26

**Authors:** Xingtai Huang, Qirui Wang, Runyi Mao, Zeying Wang, Steve G.F. Shen, Juan Mou, Jiewen Dai

**Affiliations:** 1 Department of Oral and Cranio-Maxillofacial Surgery, Shanghai Ninth People’s Hospital, Shanghai Jiao Tong University School of Medicine; College of Stomatology, Shanghai Jiao Tong University; National Center for Stomatology; National Clinical Research Center for Oral Diseases; Shanghai Key Laboratory of Stomatology, No. 639, Zhizaoju Road, 200011 Shanghai, China; 2 Department of Plastic and Reconstructive Surgery, Shanghai Ninth People’s Hospital, Shanghai Jiao Tong University School of Medicine, Shanghai, 200011 China; 3 Shanghai University of Medicine and Health Sciences, Shanghai, 201318 China; 4 The Key Laboratory of Resource Chemistry of Ministry of Education, Shanghai Key College of Chemistry and Materials Science, Shanghai Normal University, Shanghai, 200234 China

**Keywords:** Electrospun fibers, Vermiculite nanosheets, Diabetic wound healing, Angiogenesis, Composite scaffolds

## Abstract

**Background:**

Promoting diabetic wound healing is still a challenge, and angiogenesis is believed to be essential for diabetic wound healing. Vermiculite is a natural clay material that is very easy to obtain and exhibits excellent properties of releasing bioactive ions, buffering pH, adsorption, and heat insulation. However, there are still many unsolved difficulties in obtaining two-dimensional vermiculite and using it in the biomedical field in a suitable form.

**Results:**

In this study, we present a versatile organic–inorganic composite scaffold, which was constructed by embedding two-dimensional vermiculite nanosheets in polycaprolactone electrospun fibers, for enhancing angiogenesis through activation of the HIF-1α signaling pathway and promoting diabetic wound healing both in vitro and in vivo.

**Conclusions:**

Together, the rational-designed polycaprolactone electrospun fibers-based composite scaffolds integrated with two-dimensional vermiculite nanosheets could significantly improve neo-vascularization, re-epithelialization, and collagen formation in the diabetic wound bed, thus promoting diabetic wound healing. This study provides a new strategy for constructing bioactive materials for highly efficient diabetic wound healing.

**Graphical Abstract:**

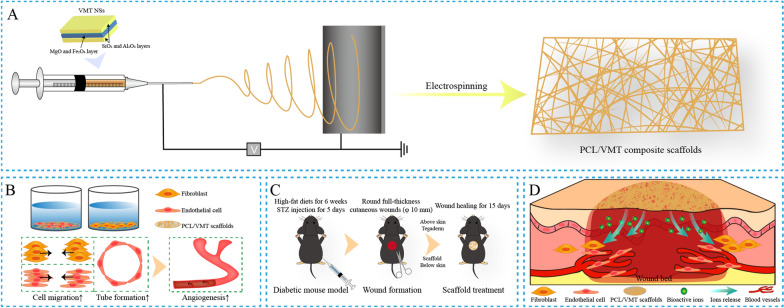

**Supplementary Information:**

The online version contains supplementary material available at 10.1186/s12951-022-01556-w.

## Introduction

Skin serves as the body’s first line of protection against external environmental elements such as physical, chemical, and biological stimuli [1]. One of the most harmful outcomes of diabetes mellitus is a persistent and non-healing skin wound [2]. Diabetic wounds are characterized by vascular impairment and delayed wound healing due to the long-term, unfavorable stimulation of high glucose, as opposed to the rapid and systematic healing processes of normal cutaneous wounds [3]. Cells and tissues are deprived of oxygen and nutrients when there is a lack of vasculature and blood circulation [4], and adequate vascularization through a tissue engineering approach may be a possible choice for promoting wound healing [5, 6].

The incorporation of clay minerals for promoting wound healing formulations is supported by its excellent biocompatibility with various skin cell types in previous studies [7, 8]. As a natural clay material, vermiculite (VMT), which exhibits the properties of releasing ions, buffering pH, adsorption, and heat insulation, has been widely used in a variety of applications, such as theranostics [9], energy resource engineering [10], and environmental management [11]. VMT belongs to the 2:1 aluminosilicate family, which consists of an octahedral layer of magnesium oxide (MgO) and ferric oxide (Fe_2_O_3_) sandwiched between two identical tetrahedral layers of silicon dioxide (SiO_2_) and aluminum oxide (Al_2_O_3_). The sandwiched layers are closely connected through water or metal ions, forming the three-dimensional (3D) structures of clay particle formations. To prepare two-dimensional (2D) clay nanosheets with the sandwiched layer as a unit, various exfoliation techniques based on breaking the linkage between the sandwiched layers, such as aqueous exfoliation [12], ion-assisted aqueous exfoliation [13], and organic polymer-assisted organic solution exfoliation [14], have been developed. However, there are still many unsolved difficulties. In addition, vermiculite nanosheets (VMT NSs) can be utilized as carriers for the sustained release of bioactive ions [15]. Direct powder application, however, has the disadvantages of being difficult to fix, poor adherence, and being easily detached [16]. Thus, clay nanosheets in two dimensions need to be evenly disseminated in polymers to create hybrid composites [17]. Integrating VMT NSs and polymers together to create a bioactive organic–inorganic hybrid platform may be an effective way of accelerating skin wound healing.

The electrospun fibrous membrane has recently generated substantial interest in skin tissue regeneration due to its impressive properties, including extracellular matrix-like structure, porous linked 3D network, huge surface-to-volume ratio, and customizable surface shape field [18–20]. In a variety of studies, bioactive components such as genes, cytokines, growth factors, and other biomolecules have been incorporated into bioactive electrospun fibrous dressings to accelerate wound healing [21–23]. Unfortunately, the direct addition of ectogenous growth factors into electrospun scaffolds has a number of drawbacks, including protein instability, high costs, and unfavorable side effects [24, 25]. The VMT NSs presented in this study have superior advantages, including low cost, straightforward manufacturing procedures, consistent chemical compositions, and long-term preservation. On the other hand, polycaprolactone (PCL) is one of the most notable synthetic polymers used for electrospun matrix in the biomedical industry, owing to its biocompatibility and biodegradability [26, 27]. According to previous studies, electrospun membranes with PCL matrix are extensively explored in biomedical applications, including in drug delivery [28], cancer treatment [29], bone regeneration engineering [30], and particularly skin wound healing [31]. Thus, developing a stable 2D VMT-loaded PCL electrospun scaffold to promote chronic skin wound healing by enhancing angiogenesis offers much promise in skin tissue engineering.

**Scheme 1:**
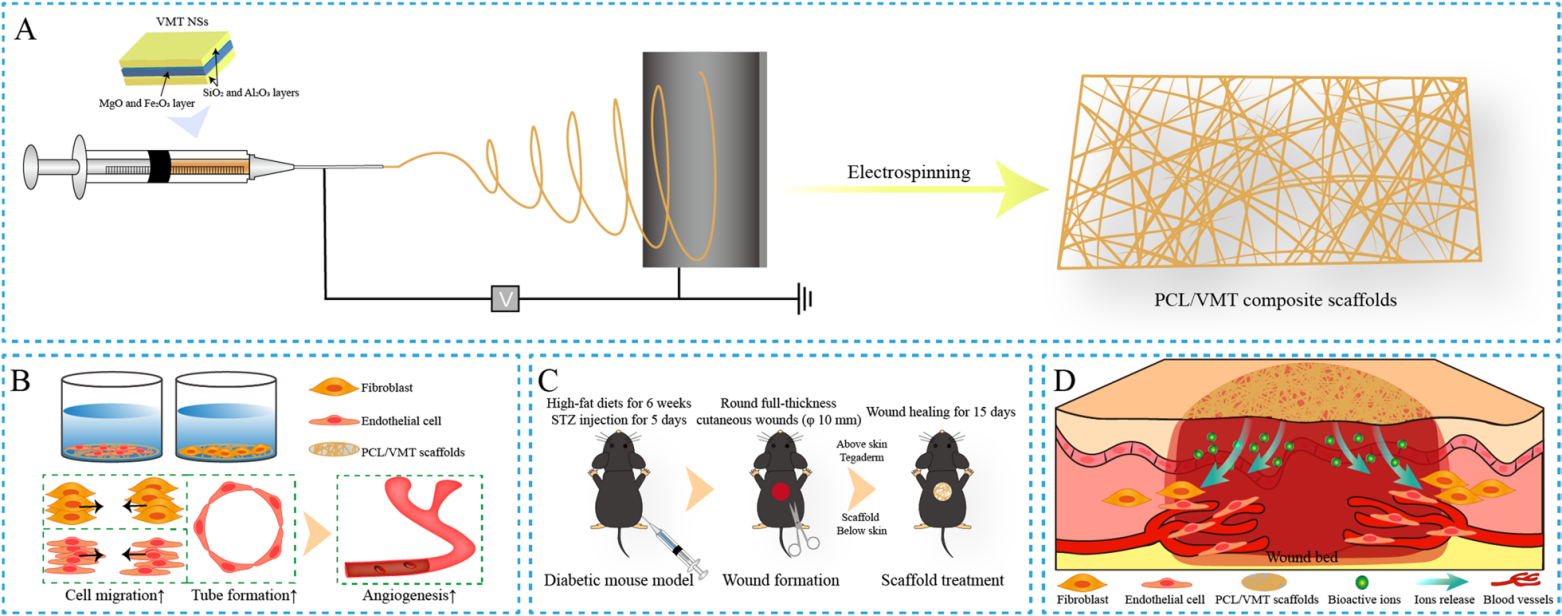
Polycaprolactone (PCL) electrospun fibers based organic–inorganic composite scaffolds integrated with two-dimension vermiculite nanosheets (VMT NSs) for diabetic wound healing. **A** Synthesis of PCL/VMT composite scaffolds. **B** In vitro evaluation of the effects of PCL/VMT composite scaffolds on wound healing-related cells. **C** In vivo evaluation of the therapeutic effect of PCL/VMT composite scaffolds for skin wound healing on a diabetic mouse model. **D** Schematic mechanism illustration of PCL/VMT composite scaffolds promoting diabetic wound healing through significantly enhanced angiogenesis, which was induced by released bioactive ions

In this study, we present a novel organic–inorganic composite scaffold constructed by integrating polycaprolactone electrospun fibers and 2D vermiculite for wound healing that promotes angiogenesis. The synthesized composite scaffolds can release bioactive ions such as silicon (Si) ions and magnesium (Mg) ions, which can promote angiogenesis [32]. Sustained expression of hypoxia-inducible factor-1α (HIF-1α) has been reported to increase angiogenesis and cutaneous wound healing in diabetic patients [33], and HIF-1α may be activated by Si ions released from the PCL/VMT scaffold, which in turn stimulates the expression of proangiogenic signaling molecules, such as vascular endothelial growth factor (VEGF) [34], endothelial nitric oxide synthase (eNOS) [35], and stromal cell-derived factor 1α (SDF-1α) [36], which can be a source of paracrine signaling among endothelial cells. As previously mentioned, it is crucial for promoting neovascularization in skin repair [37]. The as-fabricated PCL/VMT composite scaffolds could significantly promote diabetic wound healing through accelerating angiogenesis, which was systematically investigated in both in vitro cell models and in vivo diabetic wound healing mouse models (Scheme 1).

## Materials and methods

### Materials

VMT raw materials and PCL pellets were purchased from Sigma-Aldrich (USA). Hexafluoroisopropanol (HFIP) was supplied by Shanghai Macklin Biochemical Co., Ltd. (Shanghai, China). L929 (a murine fibroblast cell line) and RAW cells (RAW 264.7, a murine-derived macrophage cell line) were purchased from the Cell Bank of Shanghai Institutes for Biological Sciences (Shanghai, China). Mouse artery endothelial cells (MAECs) were purchased from FuHeng Biology (Shanghai, China). Dulbecco’s modified Eagle’s medium (DMEM), Medium 199 (M199), phosphate buffer solution (PBS), fetal bovine serum (FBS), trypsin, and penicillin-streptomycin (P/S) were purchased from Gibco (Thermo Fisher Biochemical Products Co., Ltd., USA). TRIzol, Prime Script^™^ RT Master Mix, and TB Green Premix Ex Taq were purchased from Takara Bio. Inc. (Japan). C57BL/6 (C57) male mice (6–8 weeks old; 18–22 g) were supplied by Shanghai Jihui Laboratory Animal Care Co., Ltd. (Shanghai, China).

### Preparation of VMT NSs

VMT NSs were prepared from thermally expanded vermiculite via a two-step ion-exchange method. First, bulk vermiculite granules (50 mg) were added to saturated NaCl solution (100 mL, 36 wt%) and stirred at 60 °C for 24 h under refluxing to replace the interlayer cations with Na^+^. Next, the sodium-exchanged VMT flakes were collected by filtration and washed with water and ethanol three times to remove any residual salt. Then, the as-prepared VMT was immersed in hydrogen peroxide solution (50 mL, 20 wt%) and sonicated for 30 min to exfoliate the samples into nanolayers. Finally, the exfoliated VMT nanosheets (denoted as VMT NSs) were collected by centrifugation and washed three times with deionized water for later use.

### Preparation of PCL and PCL/VMT composite scaffolds

PCL pellets (1.6 g) were weighed and dissolved in HFIP (10 mL) under magnetic stirring. Then, VMT NSs were added to the above PCL solution, and the mixture was sonicated for 30 min and continuously stirred for 12 h to obtain a homogeneous electrospinning solution. Next, composite scaffolds with various VMT NSs/PCL (W/W, 0%, 2.5%, 5%, and 10%), which were designed as the PCL, PCL/2.5%VMT, PCL/5%VMT, and PCL/10%VMT scaffolds, respectively, were fabricated by an electrostatic spinning machine. The flow rate was maintained at 1 mL/h, and the voltage was set at 14 kV. The as-synthesized composite scaffolds were dried at room temperature in a vacuum oven for 24 h to remove residual organic solvents. Finally, the scaffolds were exposed to UV irradiation for sterilization before use.

### Characterization

Transmission electron microscopy (TEM) images and energy-dispersive spectroscopy (EDS) profiles were obtained on JEOL JEM 2100 F microscope. Scanning electron microscopy (SEM) images and element mappings were acquired by Zeiss Gemini 300 microscope. Fourier transform infrared (FTIR) spectra were recorded in the range of 4000–400 cm^−1^ on an FTIR spectrometer (Thermo Scientific Nicolet iS20). X-ray diffraction (XRD) patterns were recorded on a Bruker D8 Advanced diffractometer with Cu Kα irradiation in an ambient atmosphere under constant conditions (40 kV, 40 mA, scanning range 10–65° 2θ, scanning speed 0.5°/min).

### Contact angle measurement

The water contact angle was measured on a static contact angle measuring device (SL200B, Solon Tech, China) for hydrophilicity/hydrophobicity evaluation. Briefly, the water droplet was poured onto the different scaffolds and retained for 10 s. The resulted angles between the water droplet and the surface of each specimen were photographed and recorded.

### Mechanical test

The mechanical properties of different composite scaffolds were detected by a universal mechanical tester (HY1080, Hengyi, China) using tensile mode. Samples were tailored to a size of 3 cm × 5 cm and then tested at a steady tensile speed of 5 mm/min under 500 N tension.

### Degradation evaluation

For the degradation evaluation, VMT NSs (100 mg) were immersed in phosphate-buffered saline (PBS, 10 mL, pH = 7.4) for 7 days, and the supernatant liquid was collected and exchanged at 1, 2, 3, 4, 5, 6, and 7 days. Similarly, the composite scaffolds with an average area of 2.5 × 5 cm^2^ were sealed in PBS solution (10 mL, pH = 7.4) for 14 days, and the mixture solutions were withdrawn and refreshed at 1, 2, 4, 7, 10, and 14 days. All samples were incubated at 37 °C in a shaker at a speed of 100 rpm. The supernatant was assayed by ICP for Mg and Si elemental concentration analysis, and the morphological changes of composite scaffolds were observed by SEM (Zeiss Gemini 300 microscope).

### In vitro experiments

#### Cell culture

L929 and RAW 264.7 cells were cultured in complete DMEM supplemented with 10% FBS and 1% P/S. Mouse Aortic Endothelial Cells (MAECs) were cultured in complete M199 supplemented with 10% FBS, 1% P/S, and 5 ng/mL VEGF. All cells were cultured at 37 °C in a humidified incubator with 5% CO_2_, and the medium was refreshed every 2 days.

All composite scaffolds were punched with an appropriate diameter by a hole punch to match the round shape of the cell culture plate and used for follow-up experiments after UV sterilization.

#### Cell proliferation and attachment

The proliferation of the composite scaffolds was assessed by a Cell Counting Kit-8 (CCK-8, Dojindo, Japan) assay against L929 and MAEC cells. Briefly, L929 and MAECs were seeded onto each specimen at a density of 2 × 10^4^ cells/well, respectively. CCK-8 solution (50 µL, DOJINDO Laboratories, Japan) was added to each well after 1, 3, and 5 days. The cells were incubated for another 1.5 h. The absorbance at the wavelength of 450 nm was measured on a microplate reader (Epoch, BIO-TEK, USA).

Additionally, the cell proliferation was evaluated by live-dead staining with a Calcein-AM/PI Double Staining Kit (DOJINDO laboratories, Japan). L929 and MAECs were seeded on 24-well glass-bottom culture plates (Nest Biotechnology, China) at a density of 2 × 10^5^ cells/well, respectively. Fresh culture media containing composite scaffolds extract were added. After incubation for 24 h, PBS solution containing Calcein-AM and PI was added, and the fluorescence images were acquired on a confocal laser scanning microscope (Olympus, Japan). Live and dead cells were observed at an excitation wavelength of 490 and 545 nm, respectively.

To investigate cell attachment, MAECs were seeded on the composite scaffolds in a 24-well plate at a density of 5 × 10^4^ cells/well and incubated for 24 h. The cells were then fixed with 4% paraformaldehyde (PFA) for 20 min and blocked with 1% bovine serum albumin (BSA) for 20 min. After being permeabilized in PBS solution with 0.1% Triton X-100 for 20 min, FITC-phalloidin (1:80, ABclonal Technology Co., Ltd., China) was added to label the actin microfilaments, and the cell nuclei were stained with 4,6-diamidino-2-phenylindole (DAPI, Beyotime Biotechnology, Shanghai, China). Finally, the cells were imaged with a CLSM microscope (Leica SP8, Germany).

#### Migration assay

An in vitro scratch assay was investigated to assess the migration capability of L929 and MAECs on the composite scaffolds. Defined 500 μm cell-free gaps were created by 2-well silicone inserts (ibidi, Germany). Briefly, L929 and MAECs were seeded into the culture inserts at a density of 3 × 10^5^ cells/well with a volume of 70 µL. The inserts were removed after incubation for 12 h, and the culture medium was replaced by a fresh medium containing different scaffold extracts. L929 and MAECs were fixed after incubation for 8 and 24 h, respectively, and stained with crystal violet (Beyotime Biotechnology, Shanghai, China) for 10 min. The cells were observed and photographed by inverted fluorescence microscopy. The migrated area was analyzed by Image J, and the percentage of migrated cells was quantified using the PCL group as 100%.

#### Tube formation assay

The Corning Matrigel matrix (Corning, USA) was thawed at 4 °C, added to a 24-well plate (200 µL per well), and incubated at 37 °C for 30 min. MAECs were resuspended in different extracts and seeded on Matrigel at a density of 1 × 10^5^ cells/well. After incubation for 4 h, MAECs were fixed with 4% PFA solution and stained with crystal violet for 10 min. The cells were photographed by inverted fluorescence microscopy.

#### Hemolysis test

To separate the RBCs, 1 mL of fresh rat blood was suspended in 10 mL PBS with 10 mg heparin sodium (as an anticoagulant) and centrifuged at 1000 rpm for 15 min. The cells were washed three times with PBS solutions. The PCL and PCL/VMT composite scaffolds were cut into circular films and immersed in a 0.5 mL physiological saline tube. After that, each test tube was filled with 0.5 mL of diluted blood and incubated for 1 h at 37 °C. As positive and negative controls, the same volume of RBCs solution was added to Triton X-100 (0.1%, 0.5 mL) and PBS solutions, respectively. After incubation, all samples were centrifuged for 15 min at 1000 rpm, and the absorption at 540 nm of the supernatants was measured using a microplate reader.

#### NO production

MAECs were seeded on a 24-well plate at a density of 5 × 10^4^ cells/well and incubated for 24 h before being exchanged with a fresh medium containing the composite scaffolds. NO generation in MAECs was detected by staining with the 4-amino-5-methylamino-2′,7′-difluorofluorescein diacetate (DAF-FM DA) solution (5 µM, Abcam) after incubation for 72 h. The amount of released NO in the supernatants of cell culture was determined using the Griess test. Supernatants were mixed with a Griess assay kit (Thermo Fisher, USA) to form diazonium salt, and the absorption at 540 nm was recorded on a microplate reader. The concentration of NO was determined according to the standard curve of NaNO_2_.

#### Related mRNA expression

To investigate the effect of PCL/VMT composite scaffolds on the differentiation of MAECs, gene expressions of vascular endothelial growth factor (VEGF), VEGF receptor 2 (KDR), hypoxia-inducible factor-1α (HIF-1α), basic fibroblast growth factor (bFGF), stromal cell-derived factor 1α (SDF-1α), angiopoietin-Tie receptor 2 (Tie-2), VEGF receptor 1 (Flt-1), and endothelial nitric oxide synthase (eNOS) were detected by quantitative real-time polymerase chain reaction (qRT-PCR). To detect the effect of the PCL/VMT composite scaffolds on the differentiation of L929 cells, the expressions of collagen I (Col I), collagen III (Col III), fibronectin (FN), and basic fibroblast growth factor (bFGF) were tested by qRT-PCR. The expressions of arginase (Arg) and iNOS in RAW 264.7 were also detected by qRT-PCR. All types of cells were seeded at a density of 3 × 10^5^ cells/well and a volume of 1 mL in 6-well culture plates. L929 and MAECs were cultured with composite scaffolds for 72 h, and RAW264.7 cells were cultured with composite scaffolds for 24 h. The total RNA of cells was extracted by TRIzol according to the manufacturer’s instructions. cDNA was synthesized from total RNA (800 ng) using Prime Script^™^ RT Master Mix under the conditions suggested by the manufacturer. The housekeeping gene in this experiment was glyceraldehyde-3-phosphate dehydrogenase (GAPDH). All primers were synthesized by Sangon Biotech Co., Ltd. (Shanghai, China). The primer sequences used for PCRs are presented in Additional file 1: Table S3. A LightCycler 480 PCR (Roche, Switzerland) was used to perform qRT-PCR with a volume of 20 µL SYBR Green reaction system for 50 cycles.

#### Immunofluorescence analysis

Immunofluorescence staining was performed to evaluate CD31, HIF-1α, phosphorylated endothelial nitric oxide synthase (p-eNOS), and VEGF protein expression in MAECs when cultured with different composite scaffolds. Briefly, the cells were fixed using 4% PFA for 30 min after incubation for 72 h. After being rinsed 3 times with PBS solution, cells were permeated with 0.1% Triton for 30 min. Subsequently, the cells were rinsed three times with PBS solution and then blocked with 1% BSA at room temperature for 30 min. Then, the cells were incubated with primary antibodies overnight at 4 °C. The cells were incubated with secondary antibodies for 2 h at room temperature. Phalloidin (ABclonal Technology Co., Ltd., China) and DAPI solutions (Beyotime Biotechnology, Shanghai, China) were added to the cells to counterstain the cytoskeleton and cell nuclei. Finally, the cells were photographed by laser scanning confocal microscopy. The primary antibodies used for immunofluorescences are presented in Additional file 1: Table S4. The secondary antibodies used for immunofluorescences were obtained from Jackson ImmunoResearch Inc.

#### Western blot

MAECs were cultured on composite scaffolds for 3 days, and then the total protein of MAECs was extracted by adding radioimmunoprecipitation (RIPA) lysis buffer and protease inhibitor (Beyotime Biotechnology, Shanghai, China) cocktail to plates in an ice bath. The lysates were centrifuged for 10 min with a speed of 12,000 rpm at 4 °C. Protein concentrations of the collected supernatants were determined using a BCA assay (Thermo Fisher, USA). The protein samples were separated by electrophoresis using 10% SDS polyacrylamide gels after being boiled with SDS-PAGE loading buffers (Abclonal Technology Co., Ltd., China) in a hot water bath. The proteins were then transferred to nitrocellulose membranes and treated with primary antibodies overnight at 4 °C. The primary antibodies used for western blots are presented in Additional file 1: Table S4. The membranes were subsequently treated with HRP goat anti-rabbit IgG (H+L) antibody and HRP goat anti-mouse IgG (H+L) antibody (1:2000; Abclonal Technology Co., Ltd., China) and detected by ECL reagent (Abclonal Technology Co., Ltd., China).

### In vivo experiments

#### Wound healing assessment

All animal experiments were carried out in accordance with the guidelines developed by the Institutional Animal Care and Use Committees of Shanghai Ninth People’s Hospital, and the protocols were evaluated and approved by the Ethics Committee of Shanghai Ninth People’s Hospital.

To establish the diabetes model on male C57BL/6J mice (6–8 weeks old) were given a high-fat diet for 6 weeks before receiving a daily intraperitoneal injection of streptozotocin (STZ, 50 mg/kg per day) for 5 days to cause diabetic mellitus-like symptoms. The mice were classified as diabetic if their fasting blood glucose level was over 11.1 mM for two consecutive measures and were applied for the subsequent experiments. Wounds on dorsal skin were created using a marked 10 mm punch biopsy. Different composite scaffolds (average diameter ~ 10 mm) were placed below the full-thickness excisional wound, and no implants were given to the control group. A sterile Tegaderm film (3M) was applied to protect the wound. Animals were euthanized for sample collection after healing for 15 days.

#### Histological analysis and immunofluorescence analysis

After fixation in 4% PFA, the skin samples were dehydrated in a gradient series of ethanol. For histological evaluation, the tissues were embedded in paraffin and sectioned (5 μm). Sections were deparaffinized, rehydrated, and then stained with hematoxylin and eosin (H&E) and Masson’s trichrome stain. For immunohistochemical staining of cytokeratin 10 (K10), cytokeratin 14 (K14), CD31, and endomucin (EMCN), the wound tissue sections were deparaffinized and followed a regular immunofluorescence staining process to assess re-epithelialization in wound healing. The primary antibodies used for immunofluorescences are presented in Additional file 1: Table S4. The secondary antibodies used for immunofluorescences were obtained from Jackson ImmunoResearch Inc.

#### Statistical analysis

GraphPad Prism 8.0 software was used to analyze the experimental data. All experimental data were reported as the mean ± SD (standard deviation), and each experiment has repeated a minimum of three times. Unless otherwise stated, significant differences between groups were evaluated using a one-way analysis of variance (ANOVA). When n.s. (not significant), *p < 0.05, **p < 0.01, ***p < 0.001, and ****p < 0.0001, differences were deemed statistically significant.

## Results

### Fabrication and characterization of VMT NSs and PCL/VMT composite scaffolds

**Fig. 1:**
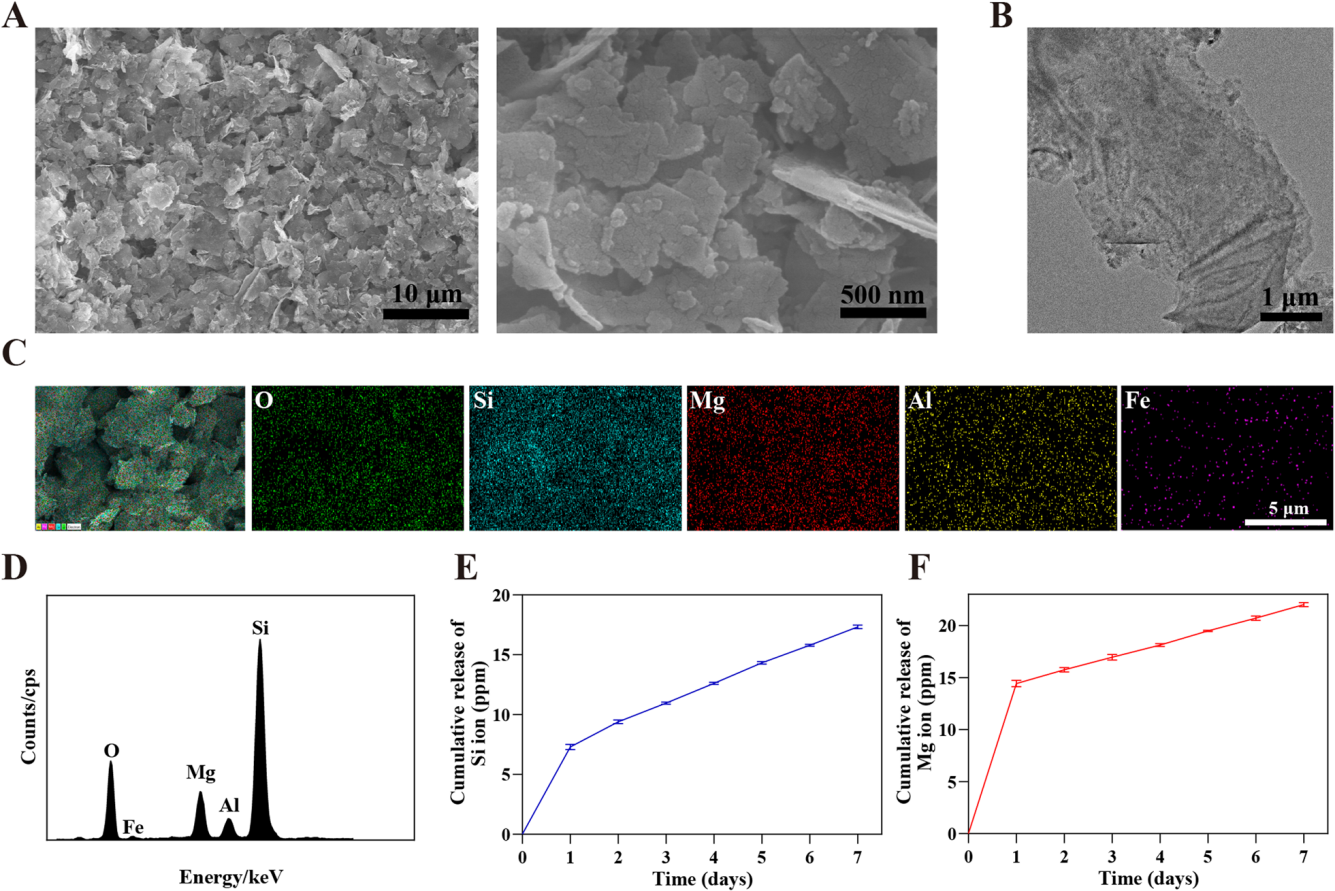
Characterization of VMT NSs. **A** SEM and **B** TEM images of VMT NSs. **C** Element-area mapping of VMT NSs. **D** EDS spectra of VMT NSs. **E**, **F** Si and Mg ions release profile of VMT NSs soaked in PBS solutions

First, the VMT NSs were facilely exfoliated through a two-step ion-exchange method from thermally expanded VMT raw materials. As shown in Fig. 1A, the SEM images of the VMT NSs displayed a typical 2D morphology of a lamellar structure with ample wrinkles and folds. A uniform morphology of the flaky structure could be observed by the TEM images of VMT NSs (Fig. 1B). The elemental mapping images (Fig. 1C) and EDS profile (Fig. 1D) confirmed the existence of the major elements, including Si, Mg, Al, Fe, and O, which were homogeneously distributed throughout the VMT NSs. As shown in Fig. 1E, F, the VMT NSs exhibited a rapid Mg and Si releasing behavior after post-incubation with PBS (pH = 7.4) on the first day and slowed down for the next 6 days.

**Fig. 2:**
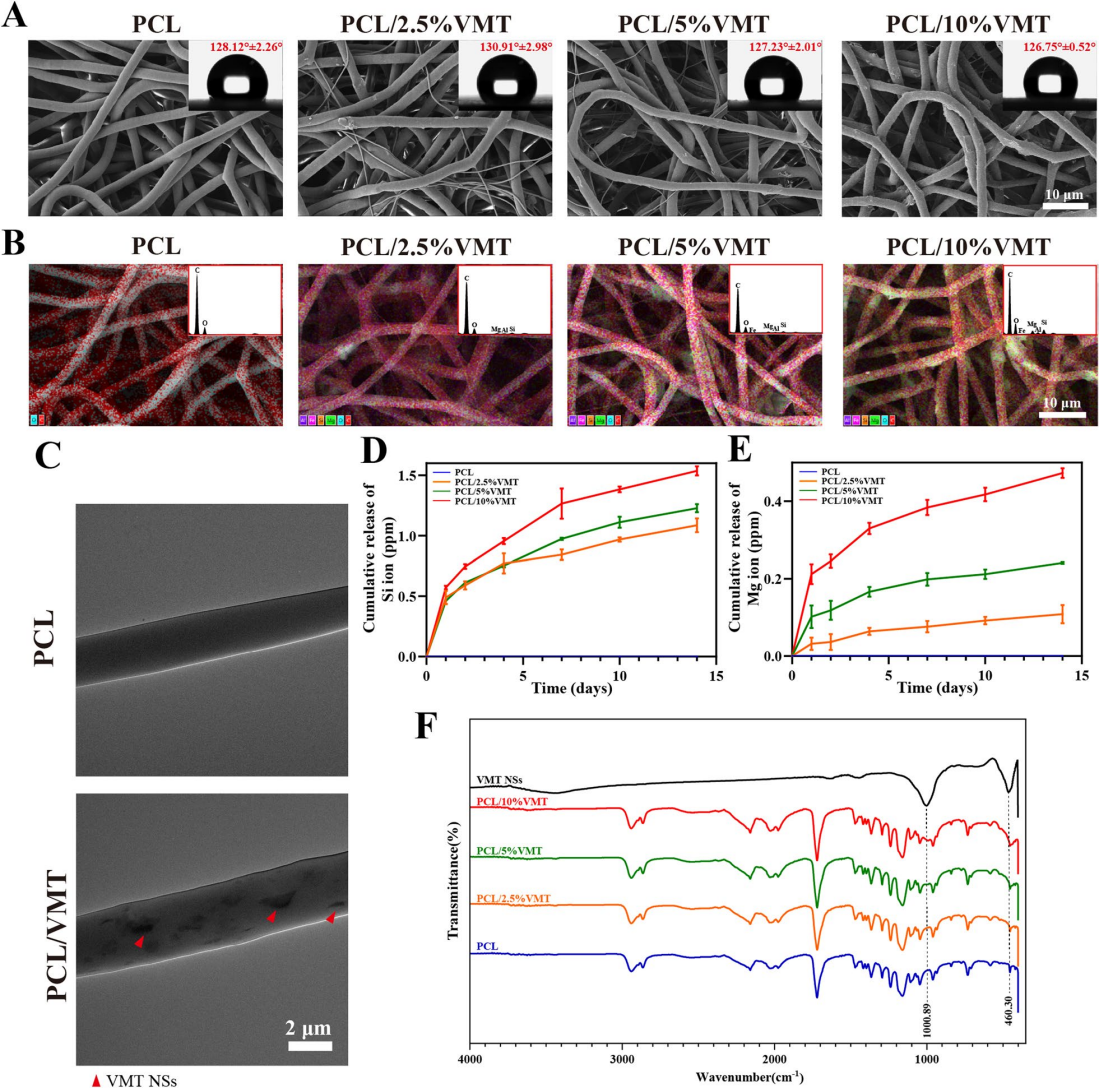
Characterization of PCL/VMT composite scaffolds. **A** SEM and water contact angle images (inset images) of PCL and PCL/VMT. **B** Element-area mapping and EDS spectra (inset images) of PCL and PCL/VMT. **C** TEM images of PCL and PCL/VMT. Red arrows point out VMT NSs. **D**, **E** Si and Mg release profile of PCL and PCL/VMT soaked in PBS solutions. **F** The FTIR spectra of VMT NSs, PCL, and PCL/VMT

Four types of PCL/VMT composite scaffolds with different weight ratios (referred to as PCL, PCL/2.5%VMT, PCL/5%VMT, PCL/10%VMT) were successfully prepared by electrospinning technology. As shown in Fig. 2A and Additional file 1: Fig. S1, the PCL scaffold displayed a smooth fibrous morphology with random alignment. After loading VMT NSs, the PCL/VMT composite scaffolds exhibited rough fibrous structures, attributing to the VMT NSs incorporation. The elemental mapping images (Fig. 2B) of PCL/VMT composite scaffolds revealed that the characteristic elements of VMT NSs, including Fe, Mg, Al, Si, and O, were distributed throughout the fibers, demonstrating the successful integration of VMT NSs, which was further confirmed by the EDS profile (inset images in Fig. 2B). TEM was used to study a single fiber of the PCL/VMT inner structure. The TEM images showed that most VMT NSs were totally encased in the fibers, and the red triangles directly indicated the existence of VMT NSs (Fig. 2C).

In addition, FTIR spectra of the PCL/VMT composite scaffolds were recorded. As illustrated in Fig. 2F, characteristic peaks around 1000 cm^−1^ and 460 cm^−1^ assigned to the ν (SiO) band and δ (SiO) band in VMT NSs [38], respectively, were clearly observed, further confirming the existence of VMT NSs in PCL/VMT composite scaffolds. Furthermore, an XRD study was performed to make sure the encapsulation of VMT into the scaffolds. As shown in Additional file 1: Fig. S2, the XRD pattern of the pure PCL scaffold displayed characteristic PCL reflections of the orthorhombic crystalline PCL structure at 21.46°, 22.23°, and 23.80°, and a relatively low and broad reflection of the semicrystalline PCL structure at 15.72° [39, 40]. The XRD pattern of VMT NSs showed reflections at 19.26°, 26.40°, 29.38°, 34.12°, 36.84°, 39.44°, 41.37°, 54.51°, and 60.11°, which belong to the phase of vermiculite-2 M (JCPDS PDF card: No. 16-0613). Comparatively, new reflections at 19.26°, 29.56°, and 40.32° appeared in the XRD pattern of the PCL/VMT composite scaffold, further indicating the VMT NSs were successfully encapsulated into the PCL scaffold. All the above-mentioned results suggested the successful fabrication of PCL/VMT composite scaffolds.

As shown in Additional file 1: Fig. S3 and Table S1, the mean diameter size of PCL, PCL/2.5%VMT, PCL/5%VMT, PCL/10%VMT composite scaffolds was 2.24 ± 0.26 μm, 2.28 ± 0.32 μm, 2.20 ± 0.34 μm and 2.23 ± 0.32 μm, respectively, indicating a negligible change in diameter size induced by the addition of VMT NSs and the uniform dispersity of VMT NSs within the fibers without aggregation.

The surface wettability of the PCL and PCL/VMT composite scaffolds was evaluated by water contact angle (WCA) measurement. As revealed by the inset images shown in Fig. 2A, the WCA values of PCL/2.5%VMT composite scaffolds were slightly increased to 130.91 ± 2.98° compared to PCL fiber with 128.12 ± 2.26°, indicating a higher hydrophobicity. However, the WCA values of the PCL/5%VMT and PCL/10%VMT composite scaffolds were successively reduced to 127.23 ± 2.01°, and 126.75 ± 0.52°, respectively, suggesting the PCL/VMT composite scaffolds preserved the similar hydrophobicity with that of PCL.

The mechanical behavior of the composite scaffolds was measured. As illustrated in Additional file 1: Fig. S4 and Table S1, the stress-strain curve of the composite scaffolds displayed a similar profile, initiated an elastic stage with a high constant stress-strain slope, and followed by a long plastic stage with a continuous reduction in the stress-strain slope toward a constant value. The tensile modulus of the composite scaffolds increased with the elevation of VMT NSs mass ratio, which increased by 97% (PCL/2.5%VMT), 117% (PCL/5%VMT), and 140% (PCL/10%VMT) compared with that of PCL, respectively, indicating the addition of VMT NSs could significantly improve the mechanical properties.

It is well-known that biomaterials will undergo degradation when exposed to biological environments and the ion release property is one of the crucial factors for evaluating biological activity. To investigate the degradation features and ion releasing, the designed PCL/VMT composite scaffolds were subjected to PBS solutions for 14 days. The concentrations of Si and Mg ions released from PCL/2.5%VMT, PCL/5%VMT, and PCL/10%VMT composite scaffolds were shown in Fig. 2D, E, respectively. The mean concentration of released Si ions increased more quickly in the first 7 days and was determined to be 0.843 ppm (PCL/2.5%VMT), 0.974 ppm (PCL/5%VMT), and 1.266 ppm (PCL/10%VMT), respectively. With the subsequently sustained release of Si ions, the mean concentration reached 1.086 ppm (PCL/2.5%VMT), 1.227 ppm (PCL/5%VMT), and 1.537 ppm (PCL/10%VMT) after incubation for 14 days. Similarly, the releasing profile of Mg showed a rapid releasing behavior in the first day with mean concentrations of 0.032 ppm (PCL/2.5%VMT), 0.102 ppm (PCL/5%VMT), and 0.212 ppm (PCL/10%VMT), respectively. Then, the release rate of Mg ions slowed down, which reached equilibrium with concentrations up to 0.108 ppm (PCL/2.5%VMT), 0.241 ppm (PCL/5%VMT), and 0.473 ppm (PCL/10%VMT) at 14 days, respectively. It could be concluded that the concentrations of released Si and Mg ions elevated with the increase of the mass of integrated VMT NSs, and the PCL/10% VMT composite scaffolds possessed the highest concentration of released Si and Mg ions compared to the other two groups. Therefore, we believed that the as-synthesized composite scaffolds could release bioactive Si and Mg ions effectively.

In addition, the morphological changes taking place to the composite scaffolds during degradation were observed by SEM. As shown in Additional file 1: Fig. S5, the mean diameter size of the PCL, PCL/2.5%VMT, PCL/5%VMT, and PCL/10%VMT was reduced to 1.93 ± 0.15 μm, 2.04 ± 0.26 μm, 1.94 ± 0.29 μm, and 2.0 ± 0.21 μm, respectively, after immersion in PBS solutions for 14 days. Compared with that of the primary composite scaffolds, the diameter size of the fibrous composite scaffolds after immersion was reduced by approximately 13.84%, 10.53%, 11.82%, and 10.31%, respectively, indicating partial degradation of the fibers. The surface of individual PCL/VMT composite scaffolds became smooth due to the detachment of VMT NSs.

### In vitro cell proliferation, attachment, and migration analysis of PCL/VMT composite scaffolds

Prior to in intro and in vivo investigation, a hemolysis test was carried out to study the hemocompatibility. As shown in Additional file 1: Fig. S6A and B, PCL, PCL/2.5%VMT, PCL/5%VMT, and PCL/10%VMT composite scaffolds displayed excellent hemocompatibility with hemolysis ratio considerably lower than 5%, within the range allowable for biomedical applications.

**Fig. 3:**
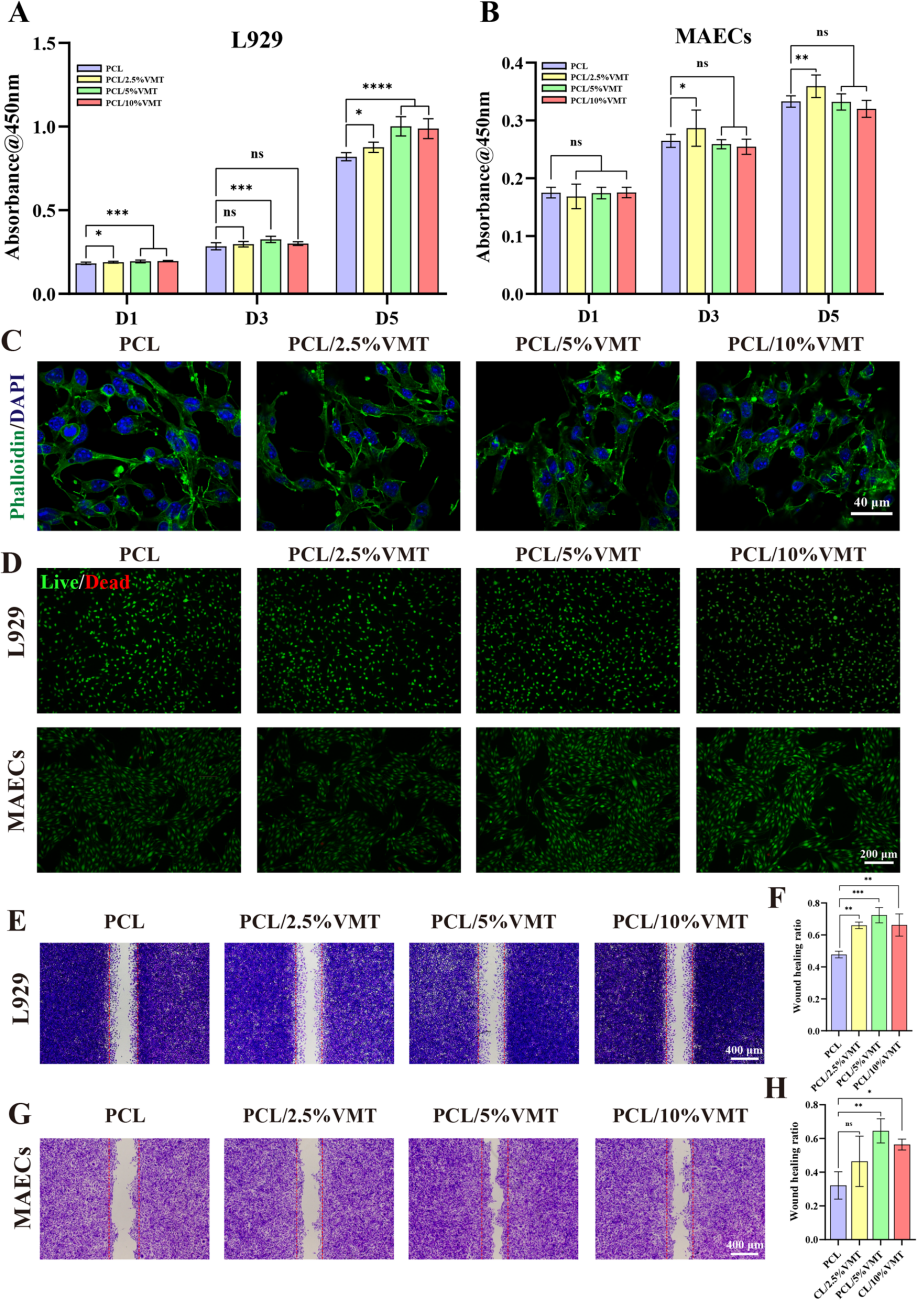
In vitro proliferation, attachment, and migration analysis of L929 and MAECs on different PCL/VMT composite scaffolds. **A**, **B** Viability of L929 and MAECs on the different scaffolds at 1, 3, and 7 days. **C** The adhesion of MAECs (green: cytoskeleton; blue: cell nuclei). **D** Live/dead staining images of L929 and MAECs after incubation for 24 h (green: live cells; red: dead cells). **E**, **F** The in vitro wound healing test of L929 cells after incubation for 8 h. **G**, **H** The in vitro wound healing test of MAECs after incubation for 24 h. Data represent means ± SD (n = 3), n.s. (not significant), *P < 0.001, **P < 0.01, ***P < 0.001, ****P < 0.001

The proliferation behaviors of L929 and MAECs on the composite scaffolds were evaluated by CCK-8 assay. Cells on four types of scaffolds exhibited significant proliferation with prolonged-incubation time (Fig. 3A, B). In addition, MAECs in all PCL and PCL/VMT composite scaffolds exhibited fusiform morphology after incubation for 24 h (Fig. 3C). Live-dead staining results further proved that the PCL/VMT composite scaffolds did not cause significant death against L929 or MAECs (Fig. 3D).

The wound-healing assay was used to analyze the migration behaviors of L929 and MAECs cultivated with different composite scaffold extracts. As shown in Fig. 3E–H, the wound healing ratio of cells in the PCL/VMT groups was higher than that of cells in the pure PCL group, and the PCL/5%VMT group exhibited the best capability to promote cell migration. These findings proved that the bioactive ions released by the PCL/VMT could promote the migration of fibroblasts and endothelial cells, and the PCL/5%VMT is the most suitable proportion. The concentration of Si ions in the PCL/5%VMT extract measured by ICP was 0.65 ± 0.03 ppm. In comparison, the cumulative concentration of Mg ions was 20.142 ± 0.004 ppm (Additional file 1: Table S2) (wherein the medium contained 19.72 ppm), which was within the biological activity concentration range of Si ions, as indicated in a previous study [41]. These findings suggested that the PCL/VMT composite scaffolds are bioactive, and bioactive ion activity may contribute to promoting endothelial cell and fibroblast viability.

### In vitro effects of PCL/VMT composite scaffolds on wound healing-related cell behavior

**Fig. 4:**
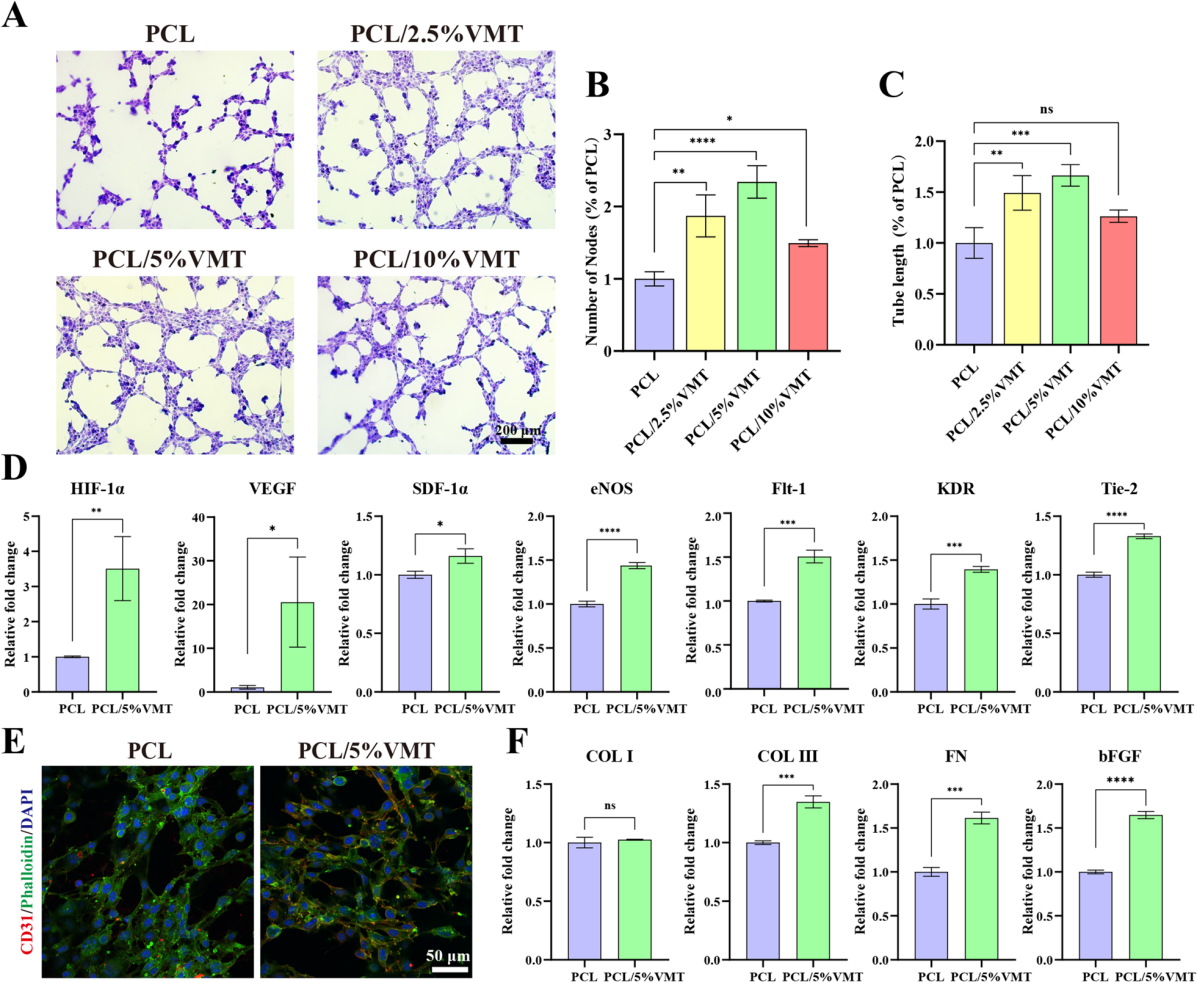
In vitro effects of PCL/VMT composite scaffolds on wound healing-related cell behavior. **A** In vitro tube formation assay of MAECs. The quantitative analysis of the number of nodes (**B**) and the tube length (**C**) formed on the Matrigel. **D** The angiogenesis-related gene expressions of HIF-1α, VEGF, SDF-1α, eNOS, Flt-1, KDR, and Tie-2 in MAECs. **E** Immunofluorescence staining of CD31, Phalloidin, and DAPI in MAECs (red: positive area of CD31; green: cytoskeleton; blue: cell nuclei). **F** The gene expressions of COL I, COL III, FN, and bFGF in L929 cells. Data represent means ± SD (n = 3), n.s. (not significant), *P < 0.001, **P < 0.01, ***P < 0.001, ****P < 0.001

The proangiogenic capacity of MAECs was evaluated using an in vitro tube formation assay. After incubation for 4 h, the MAECs in all groups formed networks of microtubule structures on the Matrigel (Fig. 4A). However, MAECs cultivated with different composite scaffolds displayed diverse self-assembly characteristics. Compared with the PCL group, the number of nodes and tube length in the PCL/VMT groups increased significantly, particularly in the PCL/5%VMT groups (Fig. 4B, C). Therefore, as a consequence of considering the findings of in vitro wound healing and tube formation assays, the PCL/5%VMT was screened out for subsequent experiments.

qPCR results showed that the expression of several key angiogenic growth factors, including HIF-1α, VEGF, SDF-1α, eNOS, Flt-1, KDR, and Tie-2, was upregulated in MAECs cultured with PCL/5%VMT compared to the PCL group (Fig. 4D), suggesting PCL/5%VMT possessed a satisfying ability to enhance MAECs angiogenic gene expression. In addition, CD31 is a vascular endothelial cell marker [42]. To confirm the vascularization ability, we used CD31 immunofluorescence staining on cells cultured with PCL/5%VMT for 72 h. The PCL/5%VMT group had higher levels of CD31 protein expression than the PCL group (Fig. 4E). According to DAF-FM fluorescence tests, ions released from the PCL/5%VMT stimulated intracellular NO production in MAECs (Additional file 1: Fig. S7A). A Griess reaction assay was used to determine the amount of NO released into the cell culture medium, and the results were consistent with those of the DAF-FM fluorescence assay (Additional file 1: Fig. S7B).

Furthermore, the expression of fibroblast genes, including collagen I (Col I), collagen III (Col III), fibronectin (FN), and basic fibroblast growth factor (bFGF), was assessed (Fig. 4F). It was found that the expression of Col III, FN, and bFGF was significantly upregulated in L929 cultured with PCL/5%VMT for 72 h compared to that with PCL. However, the expression of Col I exhibited no apparent difference between L929 cultured with PCL or PCL/5%VMT. It is well known that the normal proportions of Col I and Col III preserve the normal skin tissue structure, and a higher proportion of Col III is thought to improve the healing process and reduce scarring [43]. Our findings implied that PCL/5%VMT might have a beneficial effect on skin wound healing with fewer scars.

The gene expression of the anti-inflammatory factor ARG in RAW 264.7 cells cultured with PCL/5%VMT was considerably higher than that in RAW 264.7 cells grown with a control medium and PCL at 24 h, indicating that PCL/5%VMT might activate macrophages toward the M2 phenotype. In RAW 264.7 cells cultured with PCL/5%VMT, however, gene expression of the proinflammatory cytokine iNOS was significantly lower than in RAW 264.7 cells cultured with control media and PCL. This suggested that PCL/5%VMT could reduce proinflammatory cytokine production (Additional file 1: Fig. S8A). To further confirm our hypothesis, macrophages were treated with lipopolysaccharide (LPS), which is a prominent inducer of M1 polarization [44]. We discovered that macrophages cultured with PCL/5%VMT somewhat counteracted the effect of LPS, as evidenced by higher levels of ARG and lower levels of iNOS compared with the LPS positive control group (Additional file 1: Fig. S8B). All of the RAW 264.7 data demonstrated that PCL/5%VMT promoted macrophage polarization toward the M2 phenotype.

### In vivo PCL/VMT composite scaffolds accelerating diabetic wound healing

**Fig. 5:**
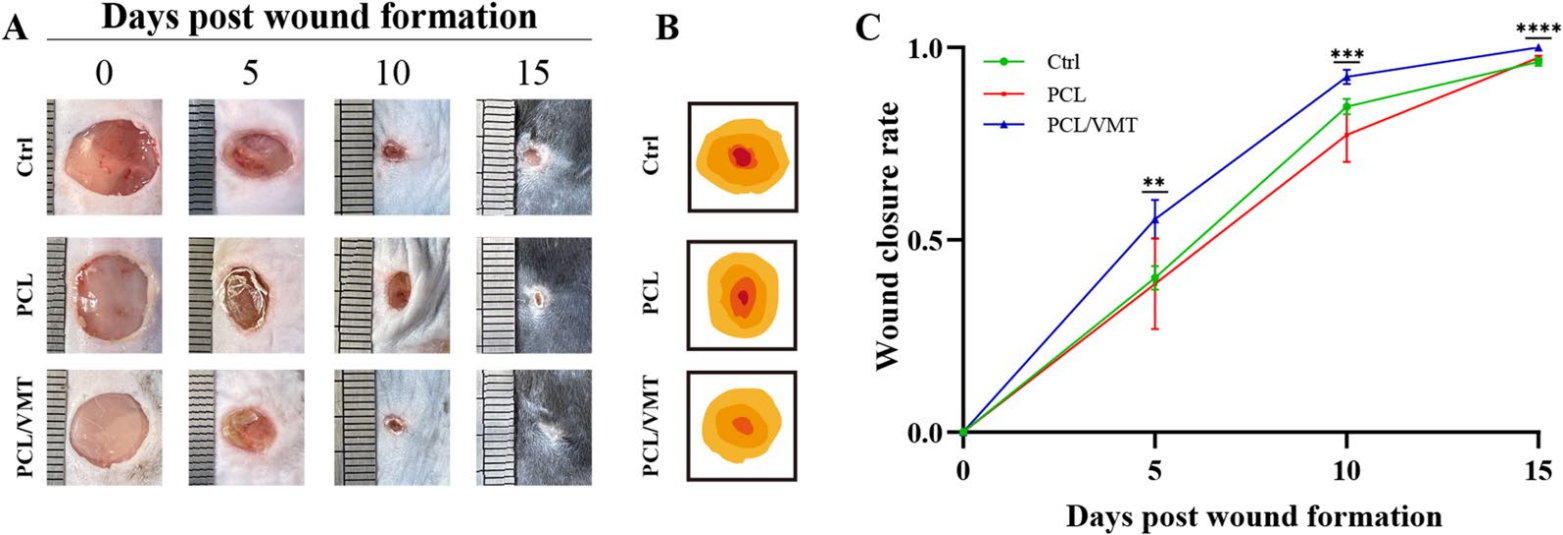
In vivo effects of PCL/5%VMT composite scaffolds on diabetic wound healing. **A** Digital images of the size change of the wounds among the three groups (control, PCL, and PCL/5%VMT) at 0, 5, 10, and 15 days post-wound formation. **B** Traces of wound bed closure for different groups in vivo. **C** Quantification of wound closure rate in each group at 0, 5, 10, and 15 days post-wound formation. Data represent means ± SD (n = 5), **P < 0.01, ***P < 0.001, ****P < 0.001

Diabetic mouse skin wounds were treated with PCL, PCL/5%VMT composite scaffolds, and the control group (uncovered). As shown in Fig. 5A, B, the wound area in all groups shrank over time. The wound closure rate of the PCL/5%VMT group was 55.4% ± 5.1% after 5 days post-wound formation, and there was a statistically significant difference (P < 0.05) when compared with the control group (40.1% ± 3.1%) and PCL group (38.5% ± 9.5%). Ten days after wound formation, the wound healing ratio in the PCL/5%VMT group was still significantly superior to that in the other two groups, and the values in the control group, PCL group, and PCL/5%VMT group were 84.6% ± 2%, 77.3% ± 5.7%, and 92.4% ± 1.9%, respectively. The PCL/5%VMT group completely healed after 15 days post-wound formation, whereas the wound healing ratio in the control group and PCL group was 96.3% ±1% and 97.4% ± 0.5% (Fig. 5C), respectively, indicating that PCL/5%VMT composite scaffolds could accelerate wound healing much more strongly than the PCL group.

**Fig. 6:**
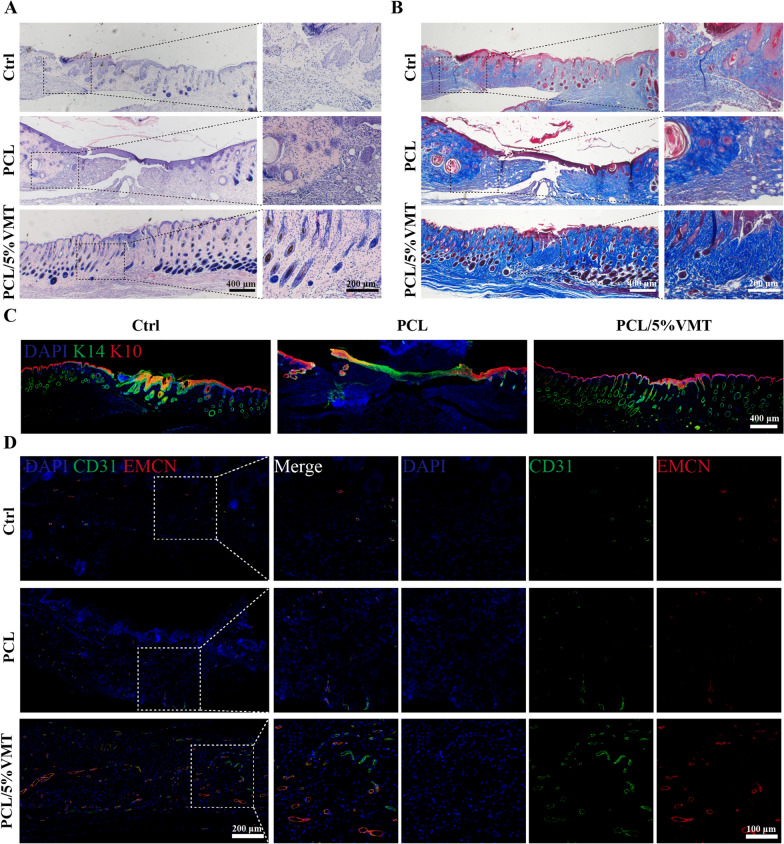
Histological staining of the wounds treated with PCL or PCL/5%VMT composite scaffolds after 15 days. **A** H&E staining. **B** Masson staining. **C** Immunofluorescence staining of K10 and K14 at the wound beds. (red: positive area of K10; green: positive area of K14; blue: cell nuclei). **D** Immunofluorescence staining of CD31 and Endomucin at the wound beds (red: positive area of EMCN; green: positive area of CD31; blue: cell nuclei)

The H&E staining results showed dermal formation in all three groups, but complete and continuous new epidermis formation was only observed in the PCL/5%VMT group (Fig. 6A). Masson’s trichrome staining results showed that the dermal layer in the PCL/5%VMT group had collagen deposition with a delicate reticular pattern and was similar to normal dermal tissues. Conversely, there was less collagen deposition in the control and PCL groups, and haphazardly structured and fibrotic deposition characteristics were observed in these two groups (Fig. 6B).

Re-epithelialization is critical in wound healing. To assess the rate of re-epithelialization, immunofluorescence staining for K10 and K14, which were two markers for spinous keratinocytes and basal keratinocytes, respectively, were performed. The results showed that basal keratinocyte migration was complete after 15 days of healing in the PCL/5%VMT group. The re-epithelialization of the PCL/5%VMT group was almost complete, and there was a distinctly stratified epithelium (Fig. 6C). Furthermore, K14 staining of the basal layer of the epidermis revealed that the hair follicle density was considerably higher in the PCL/5%VMT group than in the control and PCL groups. These findings implied that the PCL/5%VMT successfully promoted keratinocyte migration and hair follicle development, resulting in diabetic wound re-epithelialization.

The neovascularization of the three groups was examined by CD31 and EMCN immunofluorescence staining (Fig. 6D). The positive expression of CD31 and EMCN in the wound tissue of the PCL/5%VMT group was clearly higher than that of the other two groups, indicating that the addition of VMT NSs exerted a significantly active impact on stimulating angiogenesis during the wound healing process.

### Mechanism contributing to PCL/VMT promoted angiogenesis

**Fig. 7:**
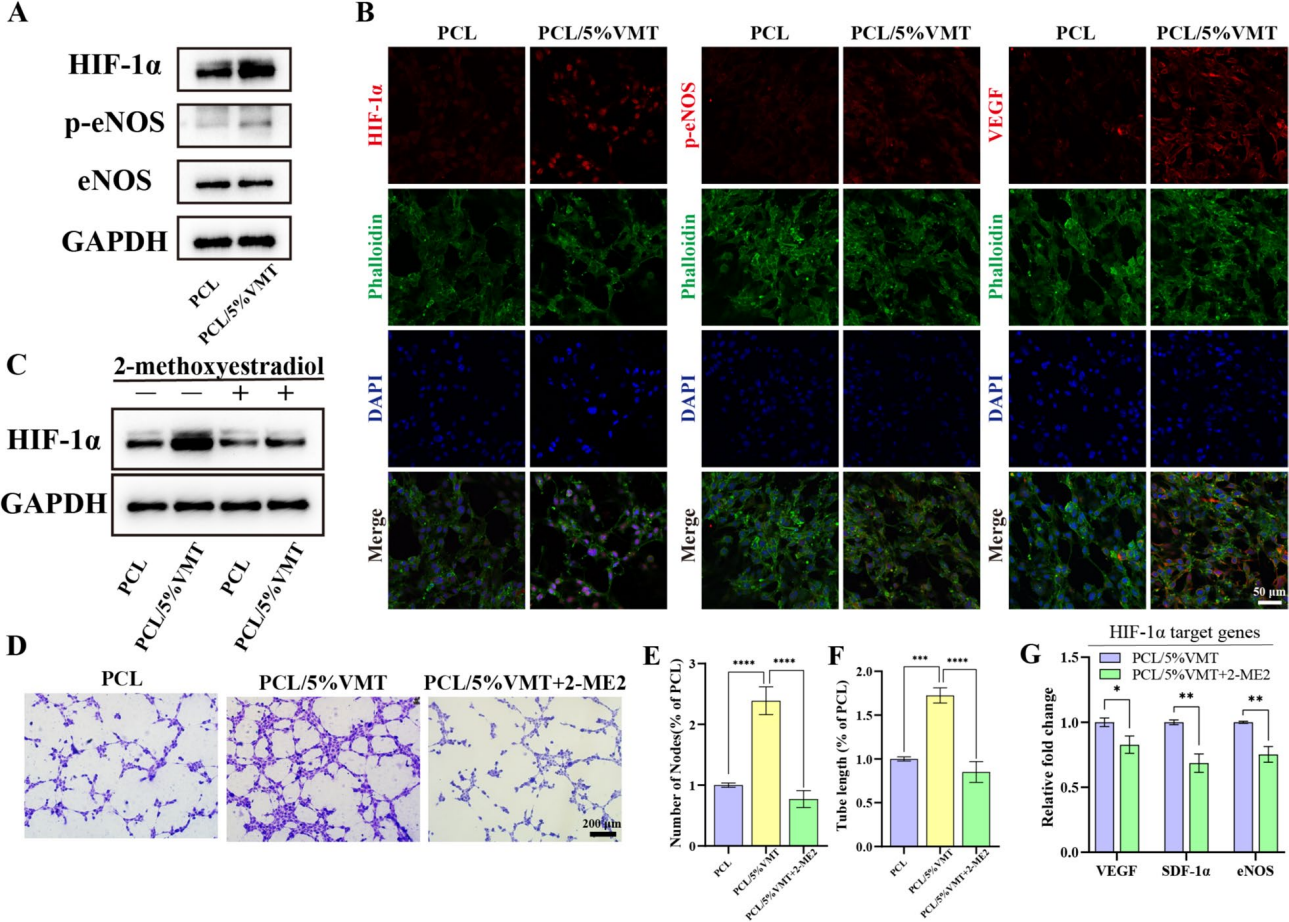
Mechanism of PCL/5%VMT promoting angiogenesis in MAECs. **A** Expression of HIF-1α, eNOS, and p-eNOS proteins. **B** Immunofluorescence staining of HIF-1α, p-eNOS, VEGF, and Phalloidin. (red: positive area of HIF-1α, p-eNOS, and VEGF; green: cytoskeleton; blue: cell nuclei). **C** Expression of HIF-1α protein and **D** in vivo tube formation assay of MAECs cultured with different composite scaffolds extracts with or without 2-ME2. The quantitative analysis of the number of nodes (**E**) and the tube length (**F**) formed on the Matrigel. **G** The gene expressions of VEGF, SDF-1α, and eNOS in MAECs cultured on PCL or PCL/5%VMT composite scaffolds with or without 2-ME2. Data represent means ± SD (n = 3), *P < 0.001, **P < 0.01, ***P < 0.001, ****P < 0.001

After culturing on PCL membranes with or without VMT NSs for 72 h, HIF-1α expression in MAECs was measured by western blot assay. The results showed that the expression of HIF-1α was upregulated in the PCL/5%VMT group compared to that of cells cultured in the pure PCL group (Fig. 7A). Phosphorylation of eNOS plays a crucial role in endothelial cell survival and angiogenesis. In this study, the expression of phosphorylated endothelial nitric oxide synthase (p-eNOS) was higher in the PCL/5%VMT group than in the pure PCL group (Fig. 7A). The immunofluorescence staining results further confirmed that the expression of HIF-1α, p-eNOS, and VEGF in MAECs was upregulated in the PCL/5%VMT group (Fig. 7B).

2-Methoxyestradiol (2-ME2) has been reported to be an effective inhibitor of HIF-1α. A concentration of 5 µM 2-ME2 was used to inhibit HIF-1α in this study. MAECs were seeded on composite scaffolds, and the cell culture media were replaced with fresh medium containing 2-ME2 or not after the cells adhered to the composite scaffolds for 24 h. The expression of HIF-1α protein in cells cultured for 48 h was assessed by western blot assay. In comparison to the PCL group, HIF-1α expression was obviously reduced in the PCL + 2-ME2 group. Meanwhile, the expression of HIF-1α was evidently suppressed in the PCL/5%VMT + 2-ME2 group as compared to the PCL/5%VMT group (Fig. 7C). These results revealed the inhibitory effect of 2-ME2 on the expression of HIF-1α. Moreover, when compared to the PCL/5%VMT group, inhibition of HIF-1α in MAECs using 2-ME2 reduced tube formation in vitro (Fig. 7D). Notably, there was no significant difference in cell angiogenesis behavior when HIF-1α was inhibited compared with the PCL group (Fig. 7E, F). In addition, the gene expression of VEGF, SDF-1α, and eNOS was downregulated when HIF-1α was inhibited (Fig. 7G). These findings indicated that PCL/5%VMT promoted angiogenesis in MAECs through a HIF-1α-dependent pathway.

## Discussion

PCL is widely used in biomaterials for tissue engineering due to its superior biocompatibility and biodegradability. However, the bioinert feature of PCL limits its potential to facilitate tissue regeneration [45]. Considerable efforts have been undertaken to increase the bioactivity of PCL grafts by coating or incorporating bioactive compounds in previous studies [46, 47]. Still, the outcomes of these modified PCL grafts have yet to be declared adequate for clinical use. As a result, new bioactive wound dressing modifications are being explored to achieve enhanced in situ skin regeneration. Herein, the concept of combining VMT NSs with PCL was proposed for promoting skin wound healing. Silicate minerals are emerging biomaterials, such as montmorillonite [48], laponite [49], and halloysite [50], which are widely used for tissue engineering. VMT is a natural silicate mineral that is frequently employed in a variety of fields owing to its simplicity of production into nanosheets. However, its application in skin tissue engineering has not been reported. In this study, we created a functional composite membrane by integrating VMT NSs into biopolymer fibers and enhancing the bioactivity of electrospun PCL membranes for skin tissue regeneration, and the results indicated the excellent property of PCL/VMT composite scaffolds for promoting skin wound healing.

Chronic wounds can be caused by diseases including diabetes, kidney infections, foreign substances, malnutrition, immunodeficiency, and advanced age, all of which affect wound healing and tissue regeneration [51]. Wound healing can be divided into the following four stages: hemostasis, inflammation, proliferation, and remodeling [1, 52]. In this study, we tried to clarify the role of PCL/VMT composite scaffolds in regulating cell proliferation for wound healing. Fibroblasts are the major cellular component of the dermis and play a key role in wound healing by secreting proteins, cytokines, and growth factors that can promote ECM production and keratinocyte function [53]. In addition, angiogenesis is crucial in the wound healing process, and previous investigations showed that poor vascularization contributed to diabetic wound healing delay. The proliferation, migration, and tube formation of endothelial cells are the main parts of the angiogenesis process [54]. In this study, the PCL/VMT composite scaffolds promoted L929 and MAEC cell adhesion and proliferation and promoted cell migration and tube formation in vitro. The PCL/VMT composite scaffolds may influence cell behavior by releasing bioactive ions because the extract of PCL/VMT composite scaffolds has a significant biological effect. In this study, Si ions reached a functional concentration that was reported in a previous study. Still, the Mg ions did not reach the concentration reported in previous literature [55], and Fe and Al ions had minimal concentrations (< 0.1 ppm), which implied that Si ions might play a crucial role in regulating cell behavior.

In this study, Col III expression was dramatically increased in L929 cells cultured with PCL/VMT. However, there was no statistical difference in the expression of Col I between L929 cells on PCL or PCL/VMT. Recent research reported that Col I plays an essential role in scar formation [56]. Meanwhile, the N-terminal propeptide of Col III retains more than that of other fibrillar collagens, suggesting its role in the control of the ECM [57]. By binding and attenuating TGFβ signaling, the CR domain within the N-propeptide of Col III improves the quality of wound healing [58]. According to our findings, PCL/VMT could significantly promote fibroblast, showing great potential to prevent scar formation during wound healing. The effect of the as-prepared PCL/VMT composite scaffolds on angiogenesis in endothelial cells showed that they could considerably upregulate the expression of angiogenesis-related genes, such as VEGF, HIF-1α, Tie-2, Flt-1, KDR, and eNOS. Importantly, stabilization of HIF-1α is vital for wound healing [59], and VEGF, SDF-1α, and eNOS are critical target genes that are regulated by HIF-1α [47, 60]. A previous study showed that the generation of eNOS causes a prolonged rise in NO, which in turn stabilizes HIF-1α protein and leads to increased HIF-1α activity in ECs [61]. Our preliminary findings suggested that eNOS may contribute to VEGF-induced angiogenesis via the intercellular messenger NO. 2-Methoxyestradiol is a HIF-1α inhibitor used in this study that could inhibit tube formation and the gene expression of VEGF and SDF-1α and further supports that the PCL/VMT composite scaffolds could promote angiogenesis by activating HIF-1α.

Newly formed blood vessels are important components of granulation tissue, and they may serve as templates for the formation of the neodermis during wound healing. There will be plenty of new capillaries to deliver nutrition and oxygen as long as angiogenesis is promoted, as well as more granulation tissue to expedite wound healing [62]. In this study, 15 days after PCL/VMT application, the regenerative vasculature could be specified as arterial and venous capillaries based on endothelial surface markers of CD31 and EMCN. Moreover, the fluorescence intensity of CD31^+^ and EMCN^+^ vessels was superior in the PCL/VMT group than in the other groups. Vessels are defined as type H (CD31^high^EMCN^high^), type E (CD31^high^EMCN^low^) and type L (CD31^low^EMCN^high^) vessels based on the expression of CD31 and EMCN. A previous study revealed type H vessels in trabecular and cortical bone next to the growth plate, as well as along the periosteal and endosteal surfaces [63], play a key role in bone regeneration [64]. In this investigation, a considerable number of type H and type E vessels were observed in the regenerated skin tissues in the PCL/VMT group, which offered full clues that selective integration of particular endothelium subtypes may be critical stages toward successful wound healing.

Electrospinning is a simple and widely accessible process for rapidly producing fibrous materials from a wide range of materials, and a multitude of ways to engineer the composition, morphology, porosity, surface, and alignment of fibers have also been reported recently [18]. Highly aligned fibers significantly impact immune microenvironments and play a regulatory role in the wound healing process [65]. In addition, the porous surface is a major physical cue that influences cell adhesion and proliferation because it can increase the effective area of membranes, which can give more active sites for cell adhesion [66]. As a nanoclay, the flake structure of VMT NSs could facilitate the assembly of proteins, peptides, and growth factors to form a slow-release system [67]. In this study, we fabricated a kind of PCL-based composite scaffolds integrated with VMT NSs. The composite scaffolds exhibited excellent performance in promoting diabetic wound healing. These findings provide new strategies for the development of nanoclay-based organic–inorganic composite biomaterials for biomedical applications.

## Conclusions

In this study, versatile PCL-based composite scaffolds incorporated with the different mass ratios of VMT NSs were successfully fabricated by electrospinning technology for promoting diabetic wound healing. The released Si and Mg ions from the scaffolds could increase the attachment, proliferation, migration, tube formation, and angiogenesis-related gene expression of MAECs. The in vivo investigation further elucidated that the as-synthesized PCL/VMT composite scaffolds could significantly improve neo-vascularization, re-epithelialization, and collagen deposition in the diabetic wound bed, resulting in eventual wound healing. The developed biocompatible and highly-effective PCL/VMT composite scaffolds hold excellent prospects for future clinical applications of skin regeneration.

## Supplementary Information

**Additional file 1: Table S1.** Mean diameter size and tensile modulus of PCL/VMT composite scaffolds. **Table S2.** Released ion concentration of Si and Mg in cell culture medium after incubation for 2 days in the presence of PCL/5%VMT scaffolds. **Table S3.** Quantitative Real-time PCR primer sequences. **Table S4.** Antibodies used for Western blot or Immunofluorescence. **Figure S1.** SEM images of the PCL, PCL/2.5%VMT, PCL/5%VMT, and PCL/10%VMT fibrous composite scaffolds. **Figure S2.** XRD patterns of PCL, PCL/VMT, and VMT NSs. **Figure S3.** Size distributions of the PCL, PCL/2.5%VMT, PCL/5%VMT, and PCL/10%VMT fibrous composite scaffolds.**Figure S4.** (A) Stress-strain curves and (B) tensile modulus ofthe PCL, PCL/2.5%VMT, PCL/5% VMT, and PCL/10%VMT composite scaffolds. **Figure S5.**(A) SEM images and (B) corresponding size distributions of the PCL, PCL/2.5%VMT, PCL/5%VMT, and PCL/10%VMT composite scaffolds immersed in PBS solutions for 14 days. **Figure S6.** In vitro hemolysis assay of the PCL, PCL/2.5%VMT, PCL/5%VMT, and PCL/10%VMT composite scaffolds. (A) Photographs and (B) absorbance of supernatants of RBCs exposed to different samples. **Figure S7.** Effects of PCL/VMT composite scaffolds on NO production in MAECs. (A) Representative fluorescent images of intracellular NO production detected by using DAF-FM probe after incubation for 72 h. (B) The amount of NO released into cell medium from MAECs cultured on the different composite scaffolds after incubation for 72 h using Griess reaction assay kits. **Figure S8.** Effects of PCL/VMT composite scaffolds on the expression of inflammatory factors in RAW 264.7. Relative mRNA expression of iNOS and Arg in the RAW 264.7 treated with PCL, PCL/2.5%VMT, PCL/5%VMT, PCL/10%VMT without (A) and with (B) LPS.

## Data Availability

All data generated or analyzed during this study are included in this published article.
