# Supplementary material for: Two-dimensional nanovermiculite and polycaprolactone electrospun fibers composite scaffolds promoting diabetic wound healing
Source: J Nanobiotechnology. 2022 Jul 26;20:343. doi: 10.1186/s12951-022-01556-w (PMC9327406; doi:10.1186/s12951-022-01556-w)
Supplement: Supplementary file 1 — Additional file 1: Table S1. Mean diameter size and tensile modulus of PCL/VMT composite scaffolds. Table S2. Released ion concentration of Si and Mg in cell culture medium after incubation for 2 days in the presence of PCL/5%VMT scaffolds. Table S3. Quantitative Real-time PCR primer sequences. Table S4. Antibodies used for Western blot or Immunofluorescence. Figure S1. SEM images of the PCL, PCL/2.5%VMT, PCL/5%VMT, and PCL/10%VMT fibrous composite scaffolds. Figure S2. XRD patterns of PCL, PCL/VMT, and VMT NSs. Figure S3. Size distributions of the PCL, PCL/2.5%VMT, PCL/5%VMT, and PCL/10%VMT fibrous composite scaffolds.Figure S4. (A) Stress-strain curves and (B) tensile modulus ofthe PCL, PCL/2.5%VMT, PCL/5% VMT, and PCL/10%VMT composite scaffolds. Figure S5.(A) SEM images and (B) corresponding size distributions of the PCL, PCL/2.5%VMT, PCL/5%VMT, and PCL/10%VMT composite scaffolds immersed in PBS solutions for 14 days. Figure S6. In vitro hemolysis assay of the PCL, PCL/2.5%VMT, PCL/5%VMT, and PCL/10%VMT composite scaffolds. (A) Photographs and (B) absorbance of supernatants of RBCs exposed to different samples. Figure S7. Effects of PCL/VMT composite scaffolds on NO production in MAECs. (A) Representative fluorescent images of intracellular NO production detected by using DAF-FM probe after incubation for 72 h. (B) The amount of NO released into cell medium from MAECs cultured on the different composite scaffolds after incubation for 72 h using Griess reaction assay kits. Figure S8. Effects of PCL/VMT composite scaffolds on the expression of inflammatory factors in RAW 264.7. Relative mRNA expression of iNOS and Arg in the RAW 264.7 treated with PCL, PCL/2.5%VMT, PCL/5%VMT, PCL/10%VMT without (A) and with (B) LPS. [file 12951_2022_1556_MOESM1_ESM.docx]

**Additional file 1**

**Two-dimensional nanovermiculite and polycaprolactone electrospun fibers composite scaffolds promoting diabetic wound healing**

Xingtai Huang^1,#^, Qirui Wang^2 ,#^, Runyi Mao^1^, Zeying Wang^1^, Steve GF Shen ^1,3^*, Juan Mou^4,^*, Jiewen Dai^1,^*

**Table S1.** Mean diameter size and tensile modulus of PCL/VMT composite scaffolds

| **Sample** | **Mean diameter size (μm)** | **Tensile modulus (kPa)** |
| --- | --- | --- |
| PCL | 2.24 ± 0.26 | 19.1 ± 3.1 |
| PCL/2.5% VMT | 2.28 ± 0.32 | 37.5 ± 3.2 |
| PCL/5% VMT | 2.20± 0.34 | 41.4 ± 2.2 |
| PCL/10% VMT | 2.23 ± 0.32 | 45.9 ± 2.4 |

**Table S2.** Released ion concentration of Si and Mg in cell culture medium after incubation for 2 days in the presence of PCL/5%VMT scaffolds

| **Element** | Si | Mg |
| --- | --- | --- |
| **Concentration (ppm)** | 0.65 ± 0.03 | 20.142 ± 0.004 |

**Table S3.** Quantitative Real-time PCR primer sequences

| Gene | Forward | Reverse |
| --- | --- | --- |
| Hif-1α | GCCAGATCTCGGCGAAGTAA | CAAATCACCAGCATCCAGAAGT |
| VEGF | TGACGGACAGACAGACAGACACC | ACGGCTACTACGGAGCGAGAAG |
| SDF-1α | GAGAGCCACATCGCCAGAG | GAGAGCCACATCGCCAGAG |
| eNOS | GTCTGCGGCGATGTCACTATGG | GTATGCGGCTTGTCACCTCCTG |
| Flt-1 | TGGACCCAGATGAAGTTCCC | GCGATTTGCCTAGTTTCAGTCT |
| KDR | GACTGAATGCGGCGGTGGTG | GTCACTGACAGAGGCGATGAATGG |
| Tie-2 | GTGTAGTGGACCAGAAGG | GTGTAGTGGACCAGAAGG |
| Col I | GGCAACAGCAGGTTCACCTACTC | GTCAGCACCACCAATGTCCAGAG |
| Col III | TCTCCTGGTGCTGCTGGTCAC | TCCATGTGGTCCAACTGGTCCTC |
| FN | ACAGTCCAGCAAGCAGCAAGC | TGGTGGTCACTCTGTAGCCTGTC |
| bFGF | AAGCGGCTCTACTGCAAGAACG | CAGCCGTCCATCTTCCTTCATAGC |
| GAPDH | AAGGTCGGTGTGAACGGATTTG | TGTAGTTGAGGTCAATGAAGGGGTC |
| Arg | CTCCAAGCCAAAGTCCTTAGAG | AGGAGCTGTCATTAGGGACATC |
| iNOS | GTTCTCAGCCCAACAATACAAGA | GTGGACGGGTCGATGTCAC |

**Table S4.** Antibodies used for Western blot or Immunofluorescence

| **Antibody** | **Source** | **Vendor** | **Catalog No.** |
| --- | --- | --- | --- |
| CD31 | Goat | R&D Systems | AF3628 |
| K10 | Rabbit | abcam | ab76318 |
| K14 | Mouse | abcam | ab7800 |
| EMCN | Rat | Santa Cruz Biotechnology | sc-65495 |
| GAPDH | Mouse | ABclonal | AC033 |
| HIF-1α | Mouse | Novus Biologicals | NB100-105 |
| eNOS | Rabbit | ABclonal | A1548 |
| p-eNOS | Rabbit | ABclonal | AP0515 |
| VEGF | Rabbit | abcam | ab52917 |


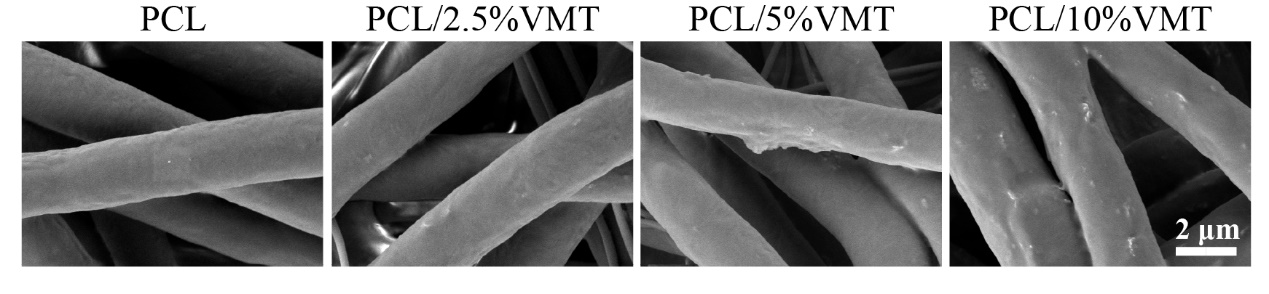


**Fig. S1.** SEM images of the PCL, PCL/2.5% VMT, PCL/5% VMT, and PCL/10% VMT fibrous composite scaffolds.


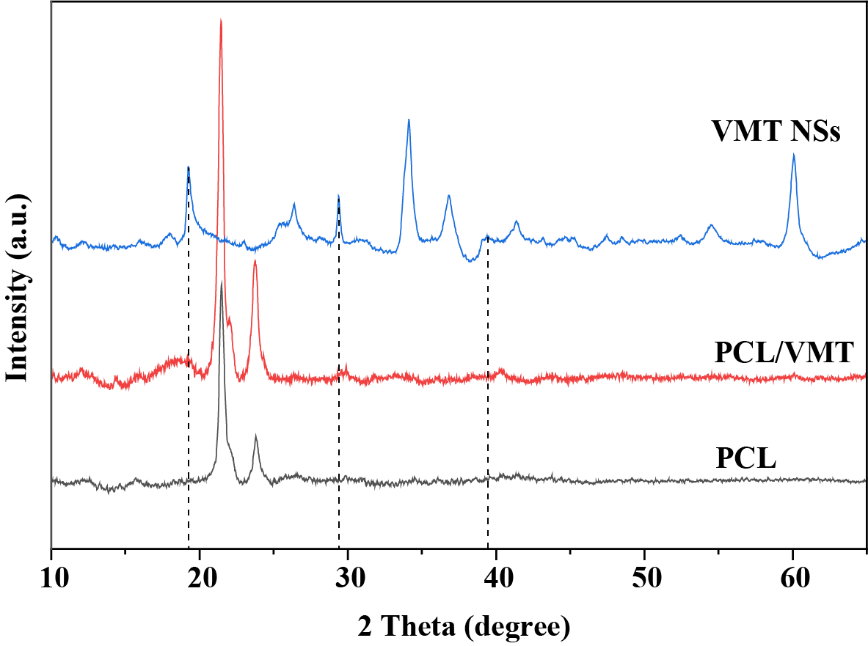


**Fig. S2.** XRD patterns of PCL, PCL/VMT, and VMT NSs.


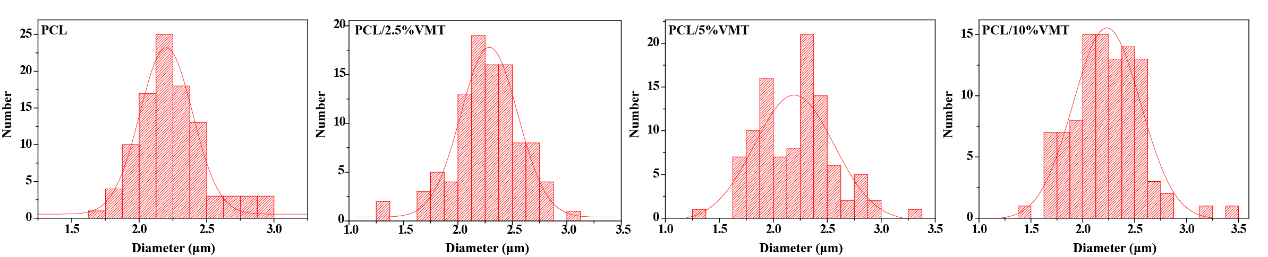


**Fig. S3.** Size distributions of the PCL, PCL/2.5% VMT, PCL/5% VMT, and PCL/10% VMT fibrous composite scaffolds.


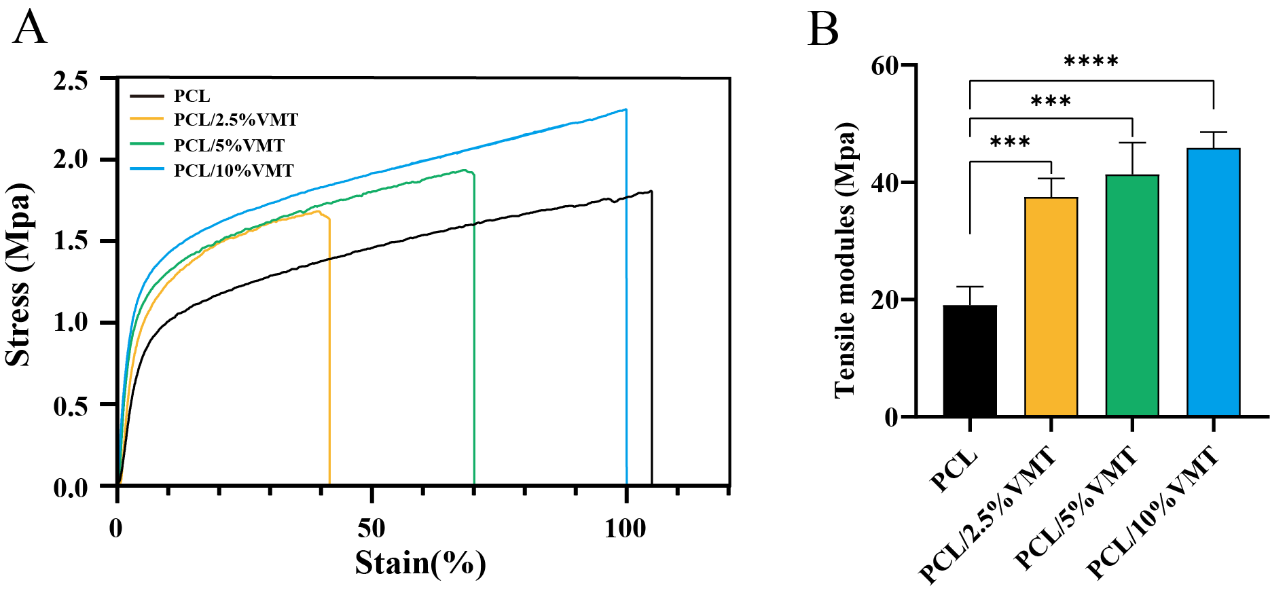


**Fig. S4.** (A) Stress-strain curves and (B) tensile modulus of the PCL, PCL/2.5% VMT, PCL/5% VMT, and PCL/10% VMT composite scaffolds.

**
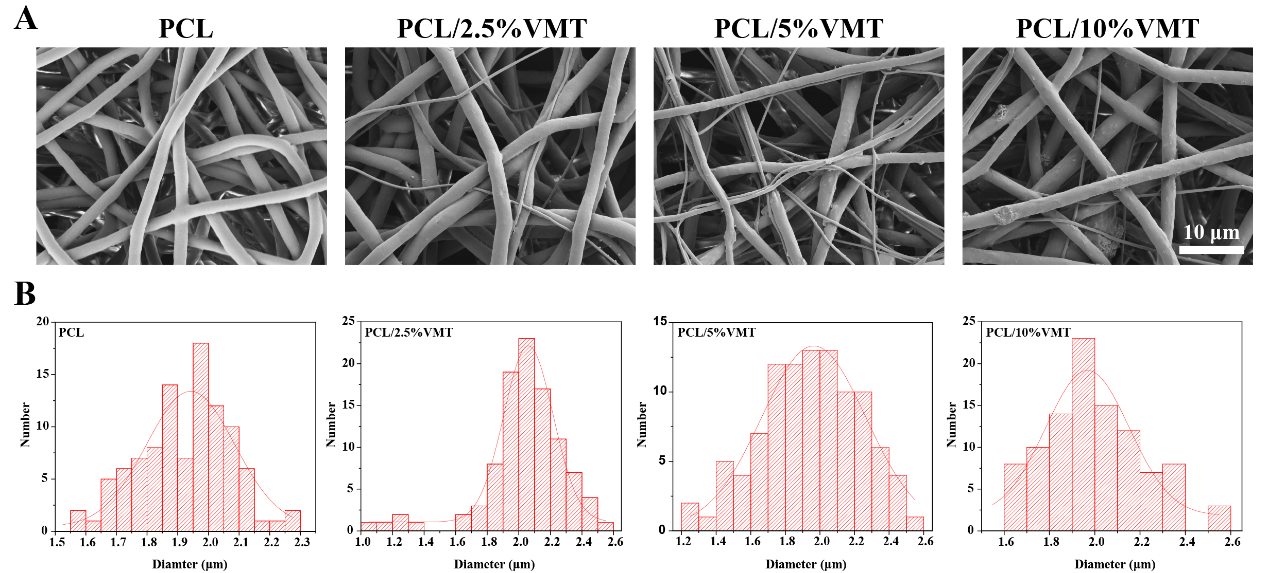
**

**Fig. S5.** (A) SEM images and (B) corresponding size distributions of the PCL, PCL/2.5% VMT, PCL/5% VMT, and PCL/10%VMT composite scaffolds immersed in PBS solutions for 14 days.


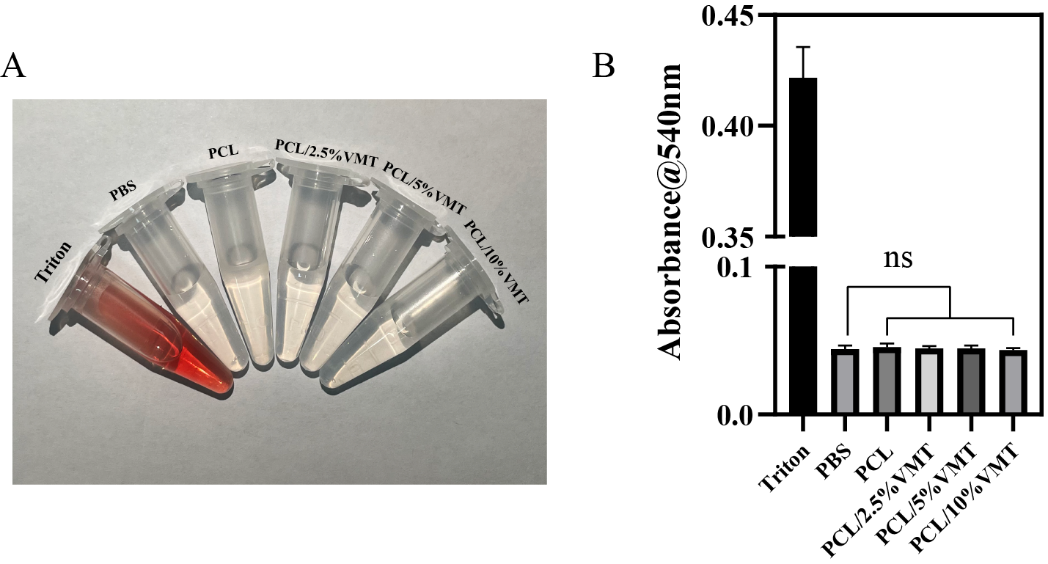


**Fig. S6.** *In vitro* hemolysis assay of the PCL, PCL/2.5% VMT, PCL/5% VMT, and PCL/10% VMT composite scaffolds. (A) Photographs and (B) absorbance of supernatants of RBCs exposed to different samples.


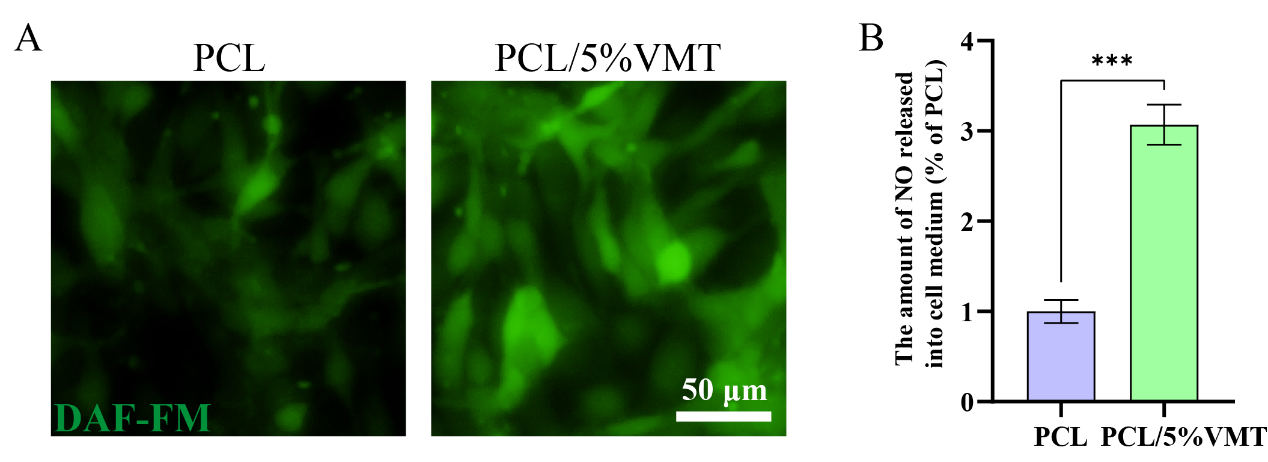


**Fig. S7.** Effects of PCL/VMT composite scaffolds on NO production in MAECs. (A) Representative fluorescent images of intracellular NO production detected by using DAF-FM probe after incubation for 72 h. (B) The amount of NO released into cell medium from MAECs cultured on the different composite scaffolds after incubation for 72 h using Griess reaction assay kits.


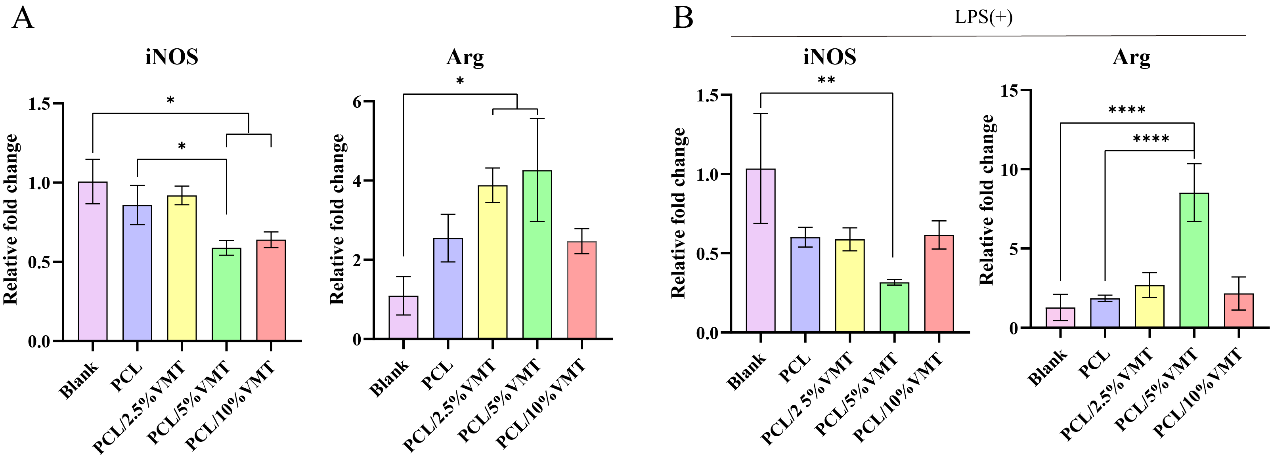


**Fig. S8.** Effects of PCL/VMT composite scaffolds on the expression of inflammatory factors in RAW 264.7. Relative mRNA expression of iNOS and Arg in the RAW 264.7 treated with PCL, PCL/2.5%VMT, PCL/5%VMT, PCL/10%VMT without (A) and with (B) [LPS](https://www.sciencedirect.com/topics/biochemistry-genetics-and-molecular-biology/lipopolysaccharide).
